# Data related to optimized process parameters influence on hardness, microstructural evolution and wear resistance performance of Al-Si-Sn-Cu/Ti-6Al-4V composite coatings

**DOI:** 10.1016/j.dib.2019.103724

**Published:** 2019-03-07

**Authors:** O.S. Fatoba, E.T. Akinlabi, S.A. Akinlabi, M.C. Obiegbu

**Affiliations:** a Department of Mechanical Engineering Science, University of Johannesburg, South Africa; b Department of Mechanical and Industrial Engineering Technology, University of Johannesburg, South Africa; c Department of Mechanical Engineering, Covenant University, Ota-Ogun State, Nigeria

**Keywords:** Ti-6Al-4V alloy, Hardness, Wear performance, Microstructure, Al-Si-Sn-Cu coatings, DLMD

## Abstract

This study investigated the metallurgical, mechanical properties and quality of coatings fabricated by direct laser metal deposition (DLMD) on Ti-6Al-4V, which were affected by the DLMD optimized process parameters. A 3-kW continuous wave ytterbium laser system (YLS) attached to a KUKA robot was used for the process. An analysis was conducted to determine the quality of the coatings in terms of hardness and wear resistance. Variables such as the time of interlayer deposition, thickness of the substrate, the initial temperature of the substrate, and the number of deposited layers were also investigated. The independent/collective effect that each process parameter had on the metallurgical and mechanical properties of the deposited Ti-6Al-4V were made clear when the processing parameters were varied. Minute pores/defects that significantly affect the metallurgical and mechanical properties of clads were also identified. The results obtained from the designed experiments showed that the depth of Heat Affected Zone (HAZ) was inversely proportional to the thickness of the substrate; as the thickness of the substrate was increased, the HAZ depth decreased. Moreover, the intensity of the laser power also affects the HAZ depth. In addition, it was discovered that the initial conditions of the substrate at room temperature also affected the coatings in relation to pre-heated conditions. The analysis conducted in identifying and quantifying the porosity showed indication that the factors such as scanning speed, laser power and powder feed rate had a predominant influence on the porosity. The grain form and structure as well as the mechanical properties of the cladded layer were significantly affected by the optimized process parameters of DLMD process. The parameters investigated had a significant impact on the hardness and wear resistance performance. Furthermore, the results revealed that the highest hardness of one of the coatings was 1.97-times the substrate which had a hardness value of 302 HV. The outstanding wear resistance performance of Al-Si-Sn-Cu/Ti-6Al-4V composite coating is attributed to major hard intermetallic phases.

**Table:** Specification table

Subject area	Materials Engineering
More specific subject area	Surface Science and Engineering
Type of data	Table and Figure
How data was acquired	Samples were characterized for SEM and energy dispersive spectroscopy (EDS) analysis. Specimens for SEM (VEGAS TESCAN) equipped with Oxford Instrument X-Max (EDS) were prepared by cutting samples in such a way to reveal the transverse section of the coatings. While Nikon optical microscope was used for the optical micrograph of the coatings. A 3-kW continuous wave (CW) Ytterbium Laser System (YLS) controlled by a KUKA robot which controls the movement of the nozzle head and emitting a Gaussian beam at 1064 nm
Experimental factors	Raw, Analyzed
Experimental features	Optimization tests were performed with the laser power of 900–1100 W and scanning speed varied from 0.6 to 1.2 m/min in order to determine the best processing parameters. The final selection criteria during optimization tests was based on surface having homogeneous layer free of porosity and cracks determined from SEM analysis. The optimum laser parameters used was 900 and 1000 W power, a beam diameter of 2 mm, gas flow rate of 2.0 L/min, powder flow rate of 2.0 g/min and scanning speeds of 1.0 and 1.2 m/min respectively.
Data source location	Department of Mechanical Engineering Science, University of Johannesburg, Johannesburg, South Africa
Data Availability	Data are available within this article.
Related research article	O.S. Fatoba, S.A. Akinlabi, E.T. Akinlabi, The effects of Sn addition on the microstructure and surface properties of laser deposited Al-Si-Sn coatings on ASTM A29 steel, IOP Conf. Ser. Mater. Sci. Eng. 328 (2018) 1–11, https://doi.org/10.1088/1757-899X/328/1/012016. M.C. Obiegbu; O.S. Fatoba; E.T. Akinlabi., S.A. Akinlabi (2019). Experimental Study on Characteristics of Laser Metal Deposited Al-Si-Sn-Cu/Ti-6Al-4V composite coatings. Materials Express Research. 6, (4), 1-11. https://doi.org/10.1088/2053-1591/aafe4d.

**Table:** 

**Value of the data** • Data presented here provide the degree of improvement achieved on each property studied depends on coating quality, refinement of the microstructures as well as the metallurgical adhesion between the coating and the substrate. The optimized process parameters used in this research resulted in huge improvement on hardness and wear resistance performance of the synthesized ternary alloy coatings. • The given data also show the proximity of the interface formed grain cells that were very small and at the regions above the scanned tracks formed larger grains that had a core of dendrite material. The high temperature and low cooling rate of the molten pool resulted in the formation of large dendrites in a solid homogenic structure which was found at a much greater laser energy density magnitude. • The given data will show author in the field of surface science and engineering, additive manufacturing the wear resistance performance of the synthesized of Al-Si-Sn-Cu Coatings on Ti-6Al-4V alloy. • The data obtained for the laser cladded Al-Si-Sn-Cu coating on Ti-6Al-4V alloy can be used as basis in determining hardness property of Al-Si-Sn-Cu/Ti-6Al-4V composite coatings. This improvement in hardness of the quaternary coatings can be attributed to homogeneous dispersion of the particulates and also in the decrease in weight percent of the particulates. • The data can be used to examine the relationship between the laser process parameters, temperature distribution and microstructural evolution of Al-Si-Sn-Cu/Ti-6Al-4V composite coatings. This could be attributed to formation of hard intermetallic compounds (Ti_3_Al, CuTi_2_, AlSi_3_Ti_2_) by temperature gradient and solidification process. The optimized process parameters had a significant influence on the thermal gradient and cooling rate, and the few defects in the coatings. The density and the microstructure of the coatings were enhanced by optimising the process parameters to obtain a properly sized melt pool which would lead to homogeneous temperature distribution.

## 1 Data

**Fig. 1:**
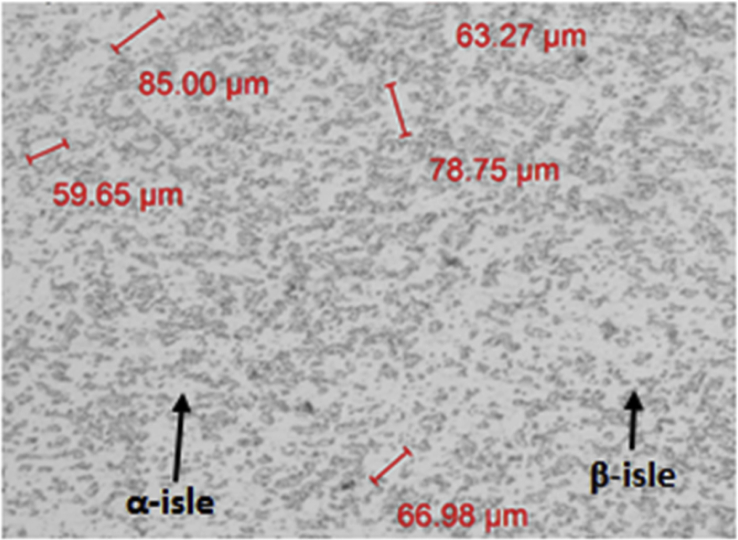
Substrate (Ti-6Al-4V) microstructure.

**Fig. 2:**
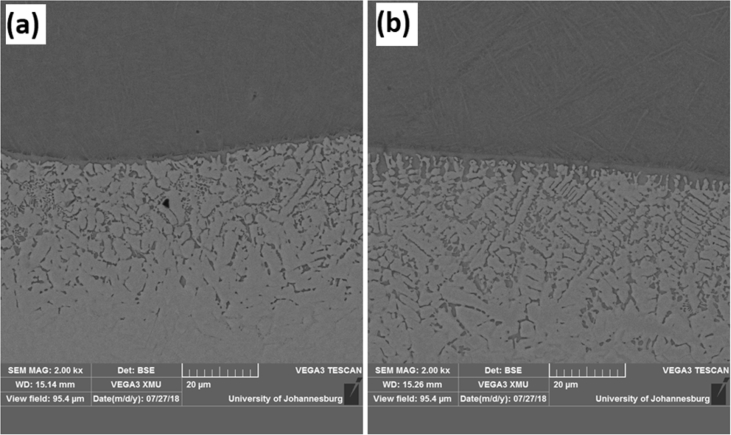
SEM images cross-section of Al-15Si-10Sn-10Cu coatings at scanning speed of 1.0 and 1.2 m/min and laser power of 1000 W.

**Fig. 3:**
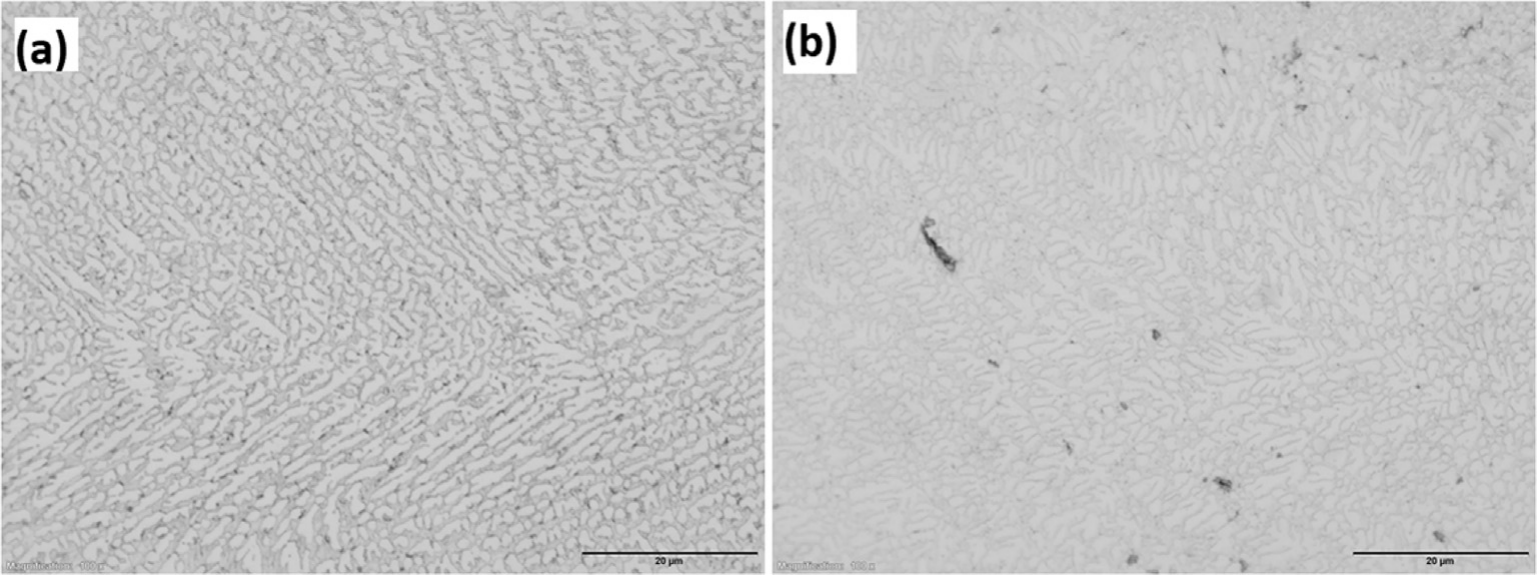
Optical micrographs of Al-15Si-10Sn-10Cu at 1.0 m/min and 1.2 m/min scanning speed and laser power of 1000 W.

**Fig. 4:**
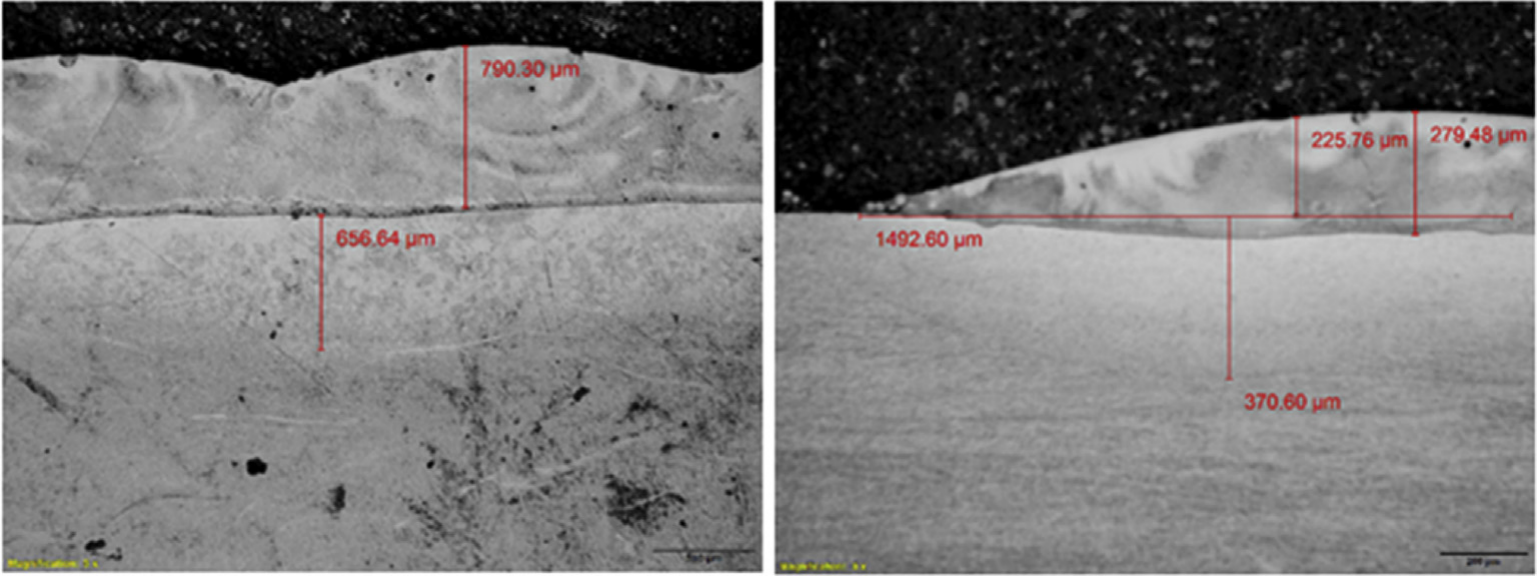
Optical micrographs cross-section of Al-15Si-10Sn-10Cu at 1.0 m/min and 1.2 m/min scanning speed and laser power of 1000 W.

**Fig. 5:**
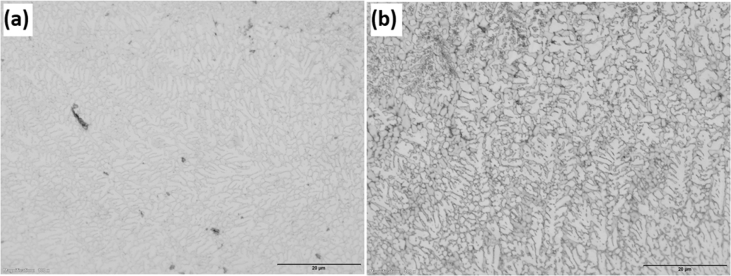
Optical micrographs of Al-15Si-10Sn-5Cu at 1.0 m/min and 1.2 m/min scanning speed and laser power of 900 W.

**Fig. 6:**
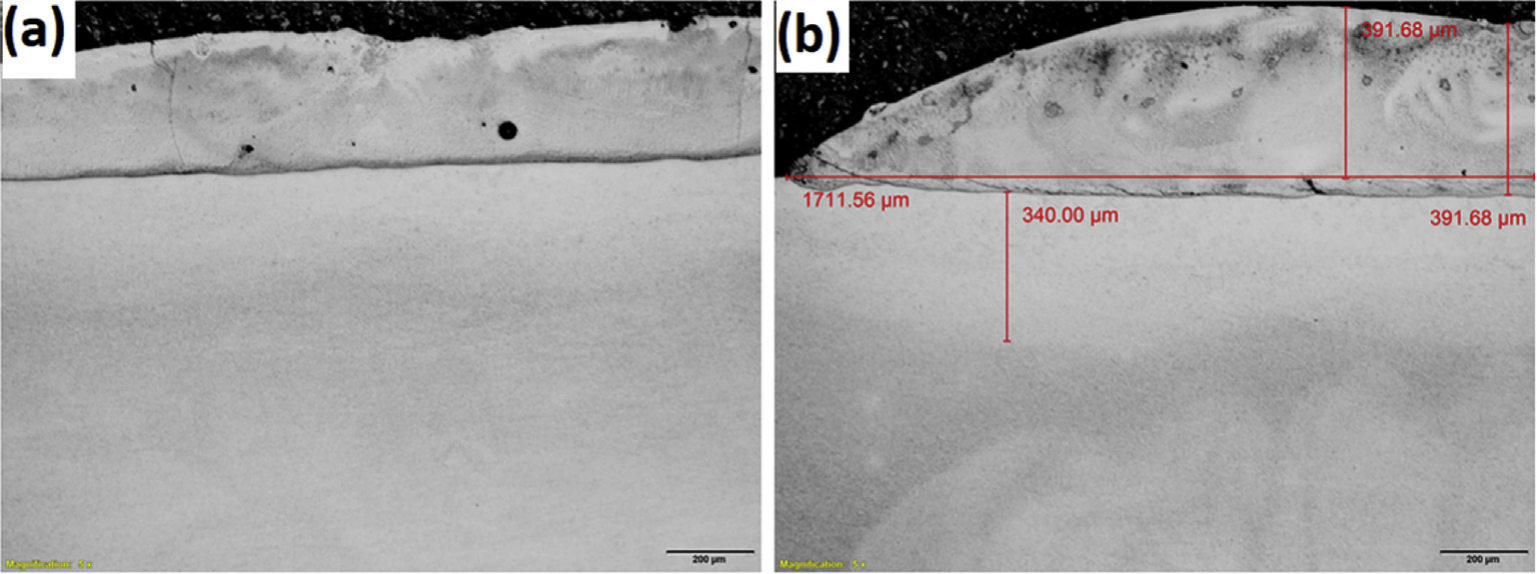
Optical micrographs cross-section of Al-15Si-10Sn-5Cu at 1.0 m/min and 1.2 m/min scanning speed and laser power of 900 W.

**Fig. 7:**
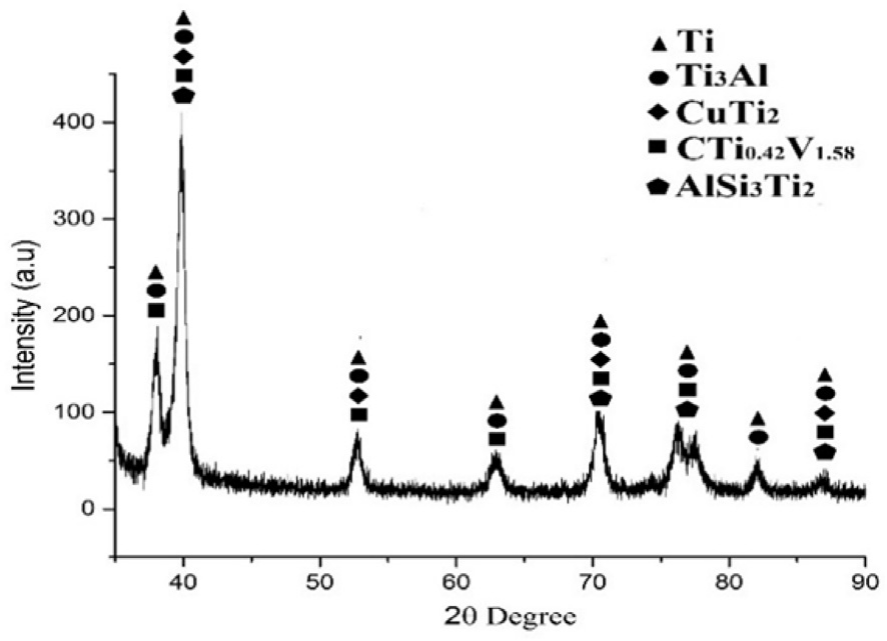
XRD spectra of Al-15Si-10Sn-10Cu Coatings at 1.2 m/min scanning speed and laser power of 1000 W.

**Fig. 8:**
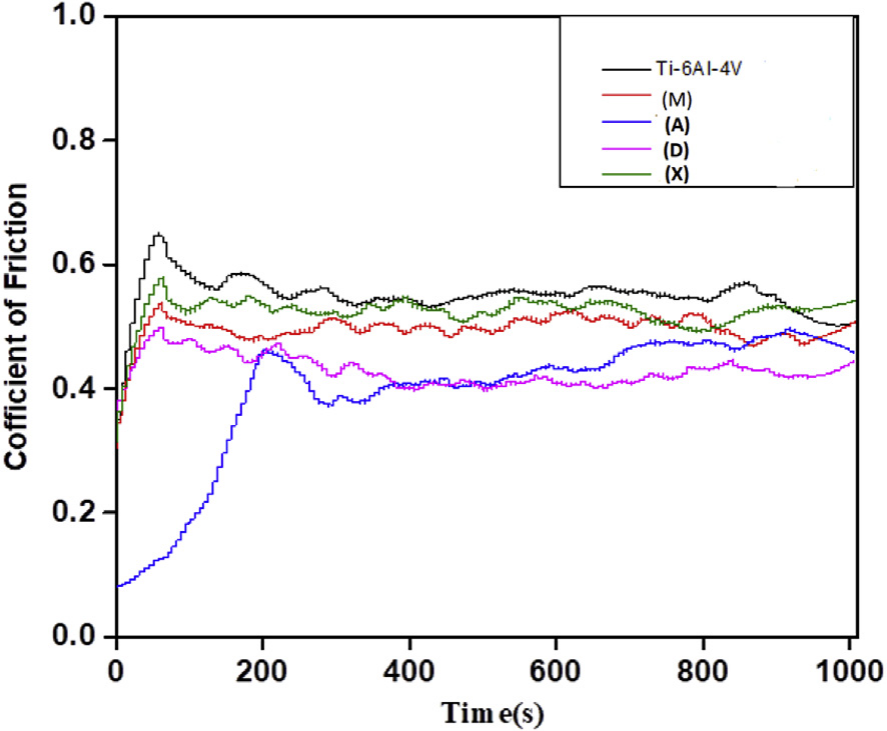
Variation of coefficients of friction of Al-Si-Sn-Cu coatings against time.

**Fig. 9:**
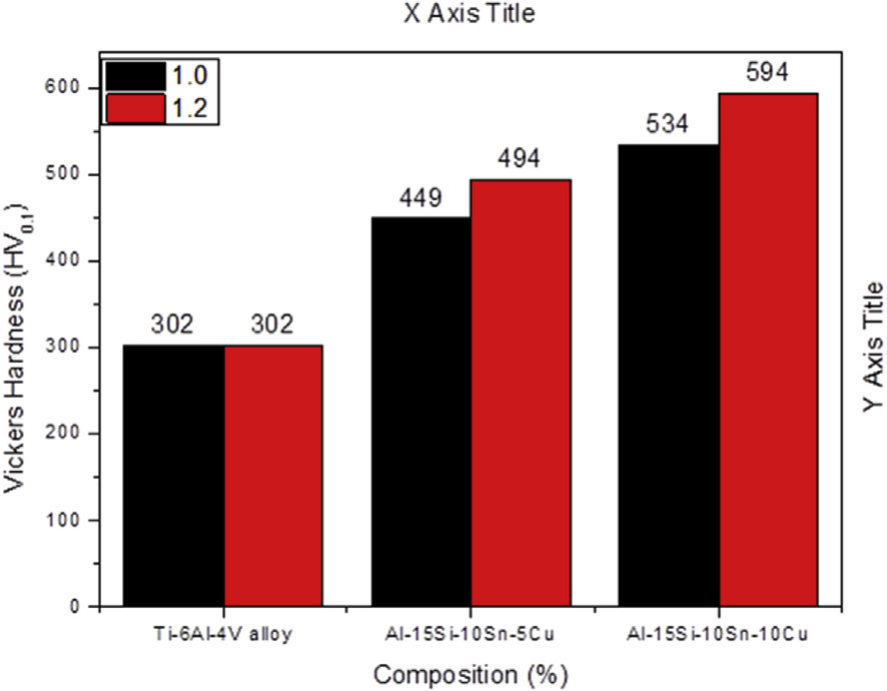
Variation of Vickers hardness of Al-Si-Sn-Cu coatings against composition.

The experimental results are presented in this session. The chemical composition of the titanium alloy grade 5 is presented in Table 1. While the microstructure of Ti-6Al-4V alloy is shown in Fig. 1. Fig. 2 shows SEM images cross-section of Al-15Si-10Sn-10Cu coatings at scanning speed of 1.0 and 1.2 m/min and laser power of 1000 W. Fig. 3 shows the optical micrographs of Al-15Si-10Sn-10Cu at 1.0 m/min and 1.2 m/min scanning speed and laser power of 1000 W. Optical micrographs cross-section of Al-15Si-10Sn-10Cu at 1.0 m/min and 1.2 m/min scanning speed and laser power of 1000 W is presented in Fig. 4. Fig. 5 shows the optical micrographs of Al-15Si-10Sn-5Cu at 1.0 m/min and 1.2 m/min scanning speed and laser power of 900 W while Fig. 6 shows the optical micrographs cross-section of Al-15Si-10Sn-5Cu at 1.0 m/min and 1.2 m/min scanning speed and laser power of 900 W. Fig. 7 presents the XRD spectrum of Al-15Si-10Sn-10Cu coatings at 1.2 m/min scanning speed and laser power of 1000 W. Variation of coefficients of friction of Al-Si-Sn-Cu coatings against time is shown in Fig. 8. Table 2 shows the Vickers hardness values of Al-Si-Sn-Cu coatings on Ti-6Al-4V alloy. Variation of Vickers hardness of Al-Si-Sn-Cu coatings against composition is shown in Fig. 9.

**Table 1:** Chemical analysis of Ti-6Al-4V substrate.

Element	Ti	Al	V	Fe	C	O	N
%Composition	Balance	6.17	3.84	0.18	0.12	0.006	0.004

**Table 2:** Hardness values of Al-Si-Sn-Cu coatings on Ti-6Al-4V alloy.

Sample number	Description	Laser Power (W)	Scanning speed (m/min)	Vickers Hardness (HV_0.1_)
Substrate	Ti-6Al-4V alloy	–	–	302.20
1 (M)	Al-15Si-10Sn-5Cu	900	1.0	448.80
2 (X)	Al-15Si-10Sn-5Cu	900	1.2	493.66
3 (A)	Al-15Si-10Sn-10Cu	1000	1.0	536.93
4 (D)	Al-15Si-10Sn-10Cu	1000	1.2	594.23

## 2 Experimental designs, materials and method

### 2.1 Materials specifications and sample preparation method

The base material used in the present investigation was Ti-6Al-4V alloy with the chemical composition shown in Table 1. 80 × 80 × 50 mm^3^ was the dimension of the base metal. Cleaning of the base metal was necessary before the starting of the DLMD process in order to reduce radiation reflection and improve the rate of absorption of beam radiation unto the substrate ref. [1], [2], [3], [4]. Aluminium with purity of 99.9%, Silicon with purity of 99.9%, Tin with purity of 99.9%, and Copper with purity of 99.9% were the reinforcements used in this research. The reinforcements particle sizes were 50–100 micron. The powders were mixed in Al-15Si-10Sn-5Cu-1.0 (Sample M), Al-15Si-10Sn-5Cu-1.2 (Sample X), Al-15Si-10Sn-10Cu-1.0 (Sample A), Al-15Si-10Sn-10Cu-1.2 (Sample D) proportions. Turbular mixer that kept the powders from oxidation and dirt was employed 14 hrs at a speed of 72 rpm in order to deliver a standardized blend of powders with dissimilar precise masses. The reinforcements are meticulously mixed in a sealed bottle ref. [5], [6], [7].

A 3-kW continuous wave (CW) Ytterbium Laser System (YLS) controlled by a KUKA robot which controls the movement of the nozzle head and emitting a Gaussian beam at 1064 nm. The robotic arm moves in a multi-axial direction and controls the laser cladding/deposition process. Admixed reinforcement materials are coaxially injected with the use of a powder feeding system fitted out with a flow balance to regulate the rate of powder flow. An argon gas flowing at a rate of 2.0 L/min was used as a protecting gas to avoid oxidation during the DLMD process ref. [8], [9]. Overlapping tracks were obtained at 50%. Cutting, grinding, and polishing were done sequentially in agreement with ASTM E3-11 standard. Optical microscopy and scanning electron microscopy were engaged to discern the microstructure of the clad in advance and afterwards the laser deposition process in agreement with ASTM F728-81 standard. X-ray Diffraction was engaged to discern the phases in the clad in agreement with ASTM E1426-14 standard. The microhardness of the base metal was measured at 302.29 HV_0.1_ in agreement with ASTM standard. The independent/collective effect that each process parameter had on the metallurgical and mechanical properties of the deposited Ti-6Al-4V were made clear when the processing parameters were varied using design of experiment. The grain form and structure as well as the mechanical properties of the cladded layer were significantly affected by the optimized process parameters of DLMD process. The laser power levels employed were 900–1000 W, powder flow rate of 2.0 g/min, gas flow rate of 2.0 L/min, a beam diameter of 2 mm, while scan velocities were 1.0–1.2 m/min.

### 2.2 Hardness property

The microhardness measurement of the alloyed samples was evaluated by using diamond indenter Vickers microhardness tester. Indentations were done at the interface at an equal dimension. The microhardness of the samples was determined with 50 μm spacing between corresponding indentations using a load of 100 gf for 10 s dwell time. Indentations were made across the deposited layer at four locations from the top of the alloyed steel to determine the hardness values at each zone. The sizes of the indentations were measured using the light optical microscope connected to the Analysis software and depth profiles of the deposited layers were determined for each sample. The average micro-hardness of all the samples was calculated using six (6) measurements obtained from different locations.

### 2.3 Wear resistance property

Tribological tests were carried out on prepared Ti-6Al-4V coatings using reciprocating UMT–2–CETR tribometer under dry sliding conditions with progressive analysis of friction coefficient values. The UMT–2–CETR tribometer equipment enables to and fro sliding action where the friction coefficients of both strokes are recorded. Specimens cut to a dimension of 2 cm × 2 cm area were fixed accurately into a sample chuck while the specimens were subjected to a vertical load in a downward motion. A normal load set at 15 N operating at a frequency of 5 Hz, sliding speed of 2 m/s, 2 mm sliding distance using tungsten carbide ball, and a period of 1000 s of reciprocating motion were used. The experimental results revealing the wear depth on the surfaces of the material and coefficient of friction were obtained using the CERT UMT-2 tribometer software.

### 2.4 Microstructural evolution

VEGA3 TESCAN (SEM) equipped with Oxford Instrument X-Max (EDS) Scanning electron microscope (SEM) was employed to investigate the morphologies of the laser clad coatings while elemental compositions were identified by energy dispersive spectroscopy (EDS). The electron acceleration voltage was varied between 15 and 20 keV. Different magnifications from 200×, 500×, 1000×, 2000×, 3000× and 5000× were used to study the microstructures of the coatings. The data collected on the sample’s surface is from certain selected areas and a 2-dimensional image is created that shows spatial differences in these properties. SEM has different magnifications that are viable between 20× to nearly 30000×, spatial resolution of 50–100 nm and it can view areas spanning from approximately 1 cm to 5 microns in width using its various modes.

### 2.5 Microstructural evolution

Nikon optical microscope was used for the optical micrograph of the coatings. Visible light is directed vertically through the microscope objective and reflected back through the objective to an eye or camera which can be used to capture the generated micrograph. The optical microscope is a type of microscope that allows magnification of the images from a size range of 500 μm to as little as 20 μm. High magnification Nikon optical micorscope was used to study the microstructure of all deposited coatings. The images of the observed microstructures were then captured with a camera fixed in the microscope. Three magnifications were used to view the detailed structures.

### 2.6 XRD spectrum

Philips PW1713 X-ray diffractometer (XRD) equipped with single colored CuKα radiation set at 40 kV and 20 mA revealed intermetallic phases present. Philips Analytical X’Pert High Scores software coupled with an in built (ICSD) database was later used for phase identification. The scan is between 10° and 80° 2 theta (2θ) and a step size of 0.02°.
