# Supplementary material for: Data related to optimized process parameters influence on hardness, microstructural evolution and wear resistance performance of Al-Si-Sn-Cu/Ti-6Al-4V composite coatings
Source: Data Brief. 2019 Mar 7;23:103724. doi: 10.1016/j.dib.2019.103724 (PMC6660619; doi:10.1016/j.dib.2019.103724)
Supplement: Supplementary file 1 — Multimedia component 1 [file mmc1.docx]

**Conflict of Interest**

No conflict of interest in this work.

Yours sincerely,


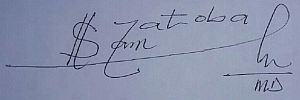


**Dr Olawale S. FATOBA. (Corresponding author).**

Department of Mechanical Engineering Science

University of Johannesburg,

South Africa.

[drfatobasameni@gmail.com](mailto:drfatobasameni@gmail.com); [proffatobasameni@gmail.com](mailto:proffatobasameni@gmail.com)

+27789549923; +27748393762.
